# A Computable Gaussian Quantum Correlation for Continuous-Variable Systems

**DOI:** 10.3390/e23091190

**Published:** 2021-09-09

**Authors:** Liang Liu, Jinchuan Hou, Xiaofei Qi

**Affiliations:** 1 College of Mathematics, Taiyuan University of Technology, Taiyuan 030024, China; liuliang@tyut.edu.cn; 2 School of Mathematical Science, Shanxi University, Taiyuan 030006, China; qixf1981@sxu.edu.cn; 3 Institute of Big Data Science and Industry, Shanxi University, Taiyuan 030006, China

**Keywords:** continuous-variable systems, Gaussian states, Gaussian geometric discord, Gaussian channels

## Abstract

Generally speaking, it is difficult to compute the values of the Gaussian quantum discord and Gaussian geometric discord for Gaussian states, which limits their application. In the present paper, for any (n+m)-mode continuous-variable system, a computable Gaussian quantum correlation M is proposed. For any state $\rho_{AB}$ of the system, $M(\rho_{AB})$ depends only on the covariant matrix of $\rho_{AB}$ without any measurements performed on a subsystem or any optimization procedures, and thus is easily computed. Furthermore, M has the following attractive properties: (1) M is independent of the mean of states, is symmetric about the subsystems and has no ancilla problem; (2) M is locally Gaussian unitary invariant; (3) for a Gaussian state $\rho_{AB}$, $M(\rho_{AB})=0$ if and only if $\rho_{AB}$ is a product state; and (4) $0\leq M\left(\left(\Phi_{A}⊗\Phi_{B}\right)\rho_{AB}\right)\leq M\left(\rho_{AB}\right)$ holds for any Gaussian state $\rho_{AB}$ and any Gaussian channels $\Phi_{A}$ and $\Phi_{B}$ performed on the subsystem A and B, respectively. Therefore, M is a nice Gaussian correlation which describes the same Gaussian correlation as Gaussian quantum discord and Gaussian geometric discord when restricted on Gaussian states. As an application of M, a noninvasive quantum method for detecting intracellular temperature is proposed.

## 1. Introduction

The presence of quantum correlations in composite quantum systems is one of the main features of quantum mechanics. Among the quantum correlations, the entanglement [1] is surely the most important one, as it is the first quantum correlation that used as physical resource. However, it is proved that non-entangled quantum correlations can also be exploited in quantum protocols. As a matter of fact, non-entangled quantum correlations not only play important roles in various quantum computing tasks and quantum communications, but also widely exist in various biological activities. In the study of photosynthesis, Cho observed quantum coherence when he investigated the energy transfer process of the Light Capture Complex by a two-dimensional spectral research method [2]. Evidence and experiments show that quantum coherence plays an important role in photosynthesis of green plants and bacteria [3,4]. By the nuclear magnetic resonance (NMR) experiments, Standish proved that nonlocal correlation exists in human brain information processing [5]. Therefore, the study and characterization of quantum correlations that go beyond the paradigm of entanglement have attracted increasingly more attention recently.

The prominent role of such quantum correlations (QCs) in the efficient realization of a number of tasks has led to the introduction of several measures of QCs. Notice that, in many quantum protocols, the systems considered are continuous variable systems. For example, the information propagated and communicated during the process of quantum communication is carried by photons, and the corresponding physical system is a continuous-variable system. For continuous-variable systems, Giorda, Paris [6] and Adesso, and Datta [7] independently proposed the definition of Gaussian quantum discord (GQD) *D* for two-mode Gaussian states $\rho_{AB}$ via the mutual information $I(\rho_{AB})$ and the extractable information $J(\rho_{AB})$ determined by performing the Gaussian positive operator value measurements (GPOVMs). It was revealed that a Gaussian state $\rho_{AB}$ contains the GQD (i.e., $D(\rho_{AB})\neq 0$) if and only if $\rho_{AB}$ is not a product state. G. Adesso and D. Girolami in [8] introduced the concept of Gaussian geometric discord (GGD) $D_{G}$ for (n+m)-mode Gaussian states $\rho_{AB}$ via GPOVMs and the Hilbert–Schmidt norm. It is also shown that $D_{G}\left(\rho_{AB}\right)=0$ if and only if $\rho_{AB}$ is a product state, that is, $\rho_{AB}$ contains no quantum correlations. Thus, both GQD and GGD are quantifications of the same bipartite Gaussian quantum correlation: $\rho_{AB}$ has the correlation if and only if $\rho_{AB}$ is not a product state. Since then, many efforts have been made to find simpler methods to quantify this Gaussian quantum correlation and various measures for it were proposed. The measurement-induced disturbance of Gaussian states was studied in [9]. Gaussian discord of response ($GD_{R}^{x}$) for two-mode Gaussian states can be found in [10]. The MIN for Gaussian states was discussed in [11]. For other related results, see in [12,13,14,15,16,17,18], and the references therein. Based on fidelity, in [19], the authors introduced a quantum correlation $N_{F}$ for Gaussian systems. The quantum non-locality $N_{F}$ for Gaussian systems is discussed in [20]. However, by now, all known quantifications of this correlation for continuous-variable systems are very difficult to compute. Most of them can only be calculated for (1+1)-mode Gaussian states or some special (n+m)-mode Gaussian states. This is mainly because all quantifications of the correlation involve measurements performed on one subsystem and optimization process, which made them difficult to evaluate. This clearly limits the applications of such Gaussian quantum correlation in real-life scenarios. Therefore, it makes sense to find simpler and computable quantifications of Gaussian quantum correlations.

According to the works in [21,22,23,24], a bona fide quantum correlation $G_{A}$ (here, local measurements are performed on subsystem A) for Gaussian states with respect to subsystem A should satisfy:

(i) $G_{A}\left(\rho_{AB}\right)=0$ if and only if $\rho_{AB}$ is a product state;
(ii) $G_{A}\left(\left(W⊗V\right)\rho_{AB}\left(W^{†}⊗V^{†}\right)\right)=G_{A}\left(\rho_{AB}\right)$ holds for any Gaussian unitary operators $W\in B(H_{A})$, $V\in B(H_{B})$ and any Gaussian state $\rho_{AB}$;
(iii) $G_{A}\left(\left(I⊗\Phi \right)\rho_{AB}\right)\leq G_{A}\left(\rho_{AB}\right)$ holds for any Gaussian channel Φ performed on subsystem B and any Guassian state $\rho_{AB}$;
(iv) There exists an entanglement measure E such that $G_{A}(|\psi \rangle \langle \psi |)=E(|\psi \rangle \langle \psi |)$ holds for any bipartite pure state |ψ〉〈ψ|.

Similar criterion should be satisfied by $G_{B}$ if local measurements are performed on subsystem B. Note that the property that $\rho_{AB}$ is a product state is symmetric about the subspace, but the quantum correlation $G_{A}$ is not in general. Therefore, it is natural and more reasonable to find Gaussian quantum correlations G that are symmetric about the subsystems and satisfy:

(a) $G(\rho_{AB})=0$ if and only if $\rho_{AB}$ is a product state;
(b) (Locally Gaussian unitary invariant) $G\left(\left(W⊗V\right)\rho_{AB}\left(W^{†}⊗V^{†}\right)\right)=G\left(\rho_{AB}\right)$ holds for any Gaussian unitary operators $W\in B(H_{A})$, $V\in B(H_{B})$ and any Gaussian state $\rho_{AB}$;
(c) (Non-increasing under local Gaussian channels) $G\left(\left(\Phi_{A}⊗\Phi_{B}\right)\rho_{AB}\right)\leq G\left(\rho_{AB}\right)$ holds for any Gaussian channels $\Phi_{A}$ and $\Phi_{B}$ performed, respectively, on subsystem A and B and any Gaussian state $\rho_{AB}$;
(d) (Reducing to an entanglement measure for pure states) There exists an entanglement measure E such that G(|ψ〉〈ψ|)=E(|ψ〉〈ψ|) holds for any bipartite pure state |ψ〉〈ψ|.

It is clear that the condition (c) implies the condition (b) and, if G satisfies properties (a–d), then it satisfies properties (i–iv).

The purpose of this paper is to propose a quantification M for bipartite Gaussian systems in terms of the covariance matrix, which avoids the measurements performed on a subsystem as well as the optimization procedure. This Gaussian correlation measure M describes the same correlation as Gaussian discord for Gaussian states but has some remarkable nice properties that the Gaussian discord does not possess: (1) M is a quantum correlation satisfying the properties (a–c), (2) M is symmetric about subsystems and has no ancilla problem, and (3) M can be estimated easily for any (n+m)-mode Gaussian states. Furthermore, M is better in detecting the non-classicality in Gaussian states as an upper bound of $N_{F}$ in [20]. Finally, as an application, we propose a noninvasive and repeatable quantum method for detecting intracellular temperature using (1+1)-mode Gaussian quantum correlation M.

## 2. Definition of the Quantity M

We first recall briefly some notions and notations concerning Gaussian states and Gaussian unitary operations. For arbitrary state ρ in a *n*-mode continuous-variable system with state space *H*, its characteristic function $\chi_{\rho }$ is defined as

$$\chi_{\rho }\left(z\right)=\mathrm{tr}\left(\rho W\left(z\right)\right),$$

where $z={\left(x_{1},y_{1},\cdots ,x_{n},y_{n}\right)}^{T}\in R^{2n}$, $W\left(z\right)=\exp\left(iR^{T}z\right)$ is the Weyl displacement operator, $R=\left(R_{1},R_{2},\cdots ,R_{2n}\right)=\left({\hat{Q}}_{1},{\hat{P}}_{1},\cdots ,{\hat{Q}}_{n},{\hat{P}}_{n}\right)$. As usual, $\hat{Q_{k}}=\left(\hat{a_{k}}+{\hat{a_{k}}}^{†}\right)/\sqrt{2}$ and $\hat{P_{k}}=-i\left(\hat{a_{k}}-{\hat{a_{k}}}^{†}\right)/\sqrt{2}$ (k=1,2,⋯,n) stand for, respectively, the position and momentum operators, where ${\hat{a}}_{k}^{†}$ and ${\hat{a}}_{k}$ are the creation and annihilation operators in the *k*th mode satisfying the Canonical Commutation Relation (CCR)

$$\left[{\hat{a}}_{k},{\hat{a}}_{l}^{†}\right]=\delta_{kl}I\;\mathrm{and}\;\left[{\hat{a}}_{k}^{†},{\hat{a}}_{l}^{†}\right]=\left[{\hat{a}}_{k},{\hat{a}}_{l}\right]=0,\;\;k,l=1,2,\cdots ,n.$$

If the state ρ has finite second-order moment, then

$$d={\left(\langle {\hat{R}}_{1}\rangle ,\langle {\hat{R}}_{2}\rangle ,\ldots ,\langle {\hat{R}}_{2n}\rangle \right)}^{T}={\left(\mathrm{tr}\left(\rho R_{1}\right),\mathrm{tr}\left(\rho R_{2}\right),\ldots ,\mathrm{tr}\left(\rho R_{2n}\right)\right)}^{T}\in R^{2n}$$

is called the mean or the displacement vector of ρ and $\Gamma =\left(\gamma_{kl}\right)\in M_{2n}\left(R\right)$ is called the covariance matrix (CM) of ρ defined by $\gamma_{kl}=\mathrm{tr}\left[\rho \left(\Delta {\hat{R}}_{k}\Delta {\hat{R}}_{l}+\Delta {\hat{R}}_{l}\Delta {\hat{R}}_{k}\right)\right]$ with $\Delta {\hat{R}}_{k}={\hat{R}}_{k}-\langle {\hat{R}}_{k}\rangle$ ([25]). Note that Γ is real symmetric and satisfies the condition Γ+iΔ≥0, where $\Delta ={⊕}_{j=1}^{n}\Delta_{j}$ with $\Delta_{j}=\left(\begin{array}{cc} 0 & 1\\ -1 & 0 \end{array}\right)$ for each *j*. Here, $M_{k}\left(R\right)$ stands for the algebra of all k×k matrices over the real field R. Denote by S(H) and FS(H), respectively, the set of all states in system *H* and the set of all states with finite second-order moment in *n*-mode CV system *H*. Moreover, ρ∈FS(H) is called a Gaussian state if $\chi_{\rho }\left(z\right)$ is of the form

$$\chi_{\rho }\left(z\right)=\exp\left[-\frac{1}{4}z^{T}\Gamma z+id^{T}z\right].$$

Now, assume that $\rho_{AB}$ is an (n+m)-mode Gaussian state with state space $H=H_{A}⊗H_{B}$. Then, the CM Γ of $\rho_{AB}$ can be written as

(1) $$\begin{array}{c} \Gamma =\left(\begin{array}{cc} A & C\\ C^{T} & B \end{array}\right), \end{array}$$

where $A\in M_{2n}\left(R\right)$, $B\in M_{2m}\left(R\right)$ and $C\in M_{2n\times 2m}\left(R\right)$. Furthermore, *A* and *B* are the CMs of the reduced states $\rho_{A}={\mathrm{tr}}_{B}\rho_{AB}$ and $\rho_{B}={\mathrm{tr}}_{A}\rho_{AB}$, respectively [26]. Actually, all the quantum correlations between subsystems *A* and *B* are embodied in *C*, to be specific, if C=0, then the Gaussian state $\rho_{AB}$ is a product state, that is, $\rho_{AB}=\sigma_{A}⊗\sigma_{B}$ for some $\sigma_{A}\in S\left(H_{A}\right)$ and $\sigma_{B}\in S\left(H_{B}\right)$[27]. Particularly, if n=m=1, by means of local Gaussian unitary (symplectic at the CM level) operations, Γ has a standard form:

(2) $$\begin{array}{c} \Gamma_{0}=\left(\begin{array}{cc} A_{0} & C_{0}\\ C_{0}^{T} & B_{0} \end{array}\right) \end{array}$$

with $A_{0}=\left(\begin{array}{cc} a & 0\\ 0 & a \end{array}\right)$, $B_{0}=\left(\begin{array}{cc} b & 0\\ 0 & b \end{array}\right)$, $C_{0}=\left(\begin{array}{cc} c & 0\\ 0 & d \end{array}\right)$, a,b≥1 and $ab-1\geq c^{2}\left(d^{2}\right)$.

For any unitary operator *U* acting on *H*, the unitary operation $\rho \mapsto U\rho U^{†}$ is said to be Gaussian if it maps Gaussian states into Gaussian states, and such *U* is called a Gaussian unitary operator. It is well known that a unitary operator *U* is Gaussian if and only if

$$\begin{array}{c} U^{†}RU=SR+m, \end{array}$$

for some vector m in $R^{2n}$ and some S∈Sp(2n,R), the symplectic group of all 2n×2n real matrices S that satisfy

$$S\in \mathrm{Sp}\left(2n,R\right)\Leftrightarrow S\Delta S^{T}=\Delta .$$

Thus, every Gaussian unitary operator *U* is determined by some affine symplectic map (S,m) acting on the phase space, and can be denoted by $U=U_{S,m}$ [26,28]. In a word, if ρ is any *n*-mode Gaussian state with CM Γ and displacement vector d, and assume that $U_{S,m}$ is a Gaussian unitary operator. Then, the characteristic function of the Gaussian state $\sigma =U_{S,m}\rho U_{S,m}^{†}$ is of the form $\exp(-\frac{1}{4}z^{T}\Gamma_{\sigma }z+id_{\sigma }^{T}z)$, where $\Gamma_{\sigma }=S\Gamma S^{T}$ and $d_{\sigma }=m+Sd$.

Now, we propose a positive function $M:\mathrm{FS}\left(H_{A}⊗H_{B}\right)\to \left[0,\infty \right)$ for continuous-variable systems in terms of the CM for (n+m)-mode states.

**Definition** **1.** *For any (n+m)-mode state $\rho_{AB}\in \mathrm{FS}\left(H_{A}⊗H_{B}\right)$ with CM $\Gamma =\left(\begin{array}{cc} A & C\\ C^{T} & B \end{array}\right)$, the quantity $M(\rho_{AB})$ is defined by*

(3) $$\begin{array}{c} M\left(\rho_{AB}\right)=1-\frac{\det(\Gamma )}{\left(\det A)(\det B\right)} \end{array}$$

Clearly, M is very easily evaluated for any (n+m)-mode state $\rho_{AB}\in \mathrm{FS}\left(H_{A}⊗H_{B}\right)$ because no measurements are involved and no optimization procedure is needed.

Definition 1 is inspired by the work in [20], in which Gaussian quantum correlation $N_{F}^{G,A}$ was introduced and discussed. For any (n+m)-mode state $\rho_{AB}\in S\left(H_{A}⊗H_{B}\right)$ with CM $\Gamma =\left(\begin{array}{cc} A & C\\ C^{T} & B \end{array}\right)$, the quantity $N_{F}^{G,A}\left(\rho_{AB}\right)$ is defined as -4.6cm0cm

$$N_{F}^{G,A}\left(\rho_{AB}\right)=\underset{U}{\sup}C^{2}\left(\rho_{AB},\left(U⊗I\right)\rho_{AB}\left(U^{†}⊗I\right)\right)=\underset{U}{\sup}\left\{1-\frac{{\left(\mathrm{tr}\rho_{AB}\left(U⊗I\right)\rho_{AB}\left(U^{†}⊗I\right)\right)}^{2}}{\mathrm{tr}\left(\rho_{AB}^{2}\right)\mathrm{tr}{\left(\left(U⊗I\right)\rho_{AB}\left(U^{†}⊗I\right)\right)}^{2}}\right\},$$

where the supremum is taken over all Gaussian unitary operators $U\in B(H_{A})$ satisfying $U\rho_{A}U^{†}=\rho_{A}$ with $\rho_{A}={\mathrm{Tr}}_{B}\rho_{AB}$ the reduced state. It was shown in [20] that, for any (n+m)-mode state $\rho_{AB}$ with CM $\Gamma =\left(\begin{array}{cc} A & C\\ C^{T} & B \end{array}\right)$, we have

(4) $$N_{F}^{G,A}\left(\rho_{AB}\right)\leq 1-\frac{\det(B-C^{T}A^{-1}C)}{\det B}.\;$$

However, it is well known that the determinant $\det\left(\begin{array}{cc} A & C\\ D & B \end{array}\right)=\left(\det A\right)\left(\det\left(B-DA^{-1}C\right)\right)$ if *A* is invertible and $\det\left(\begin{array}{cc} A & C\\ D & B \end{array}\right)=\left(\det B\right)\left(\det\left(A-CB^{-1}D\right)\right)$ if *B* is invertible (see, for example, in [29]). Thus,

$$M\left(\rho_{AB}\right)=1-\frac{\det(\Gamma )}{\left(\det A)(\det B\right)}=1-\frac{\det(B-C^{T}A^{-1}C)}{\det B}=1-\frac{\det(A-CB^{-1}C^{T})}{\det A}.$$

Therefore, M is exactly an upper bound for the Gaussian quantum correlation $N_{F}^{G,A}$ obtained in [20]. Note that the Gaussian correlation $N_{F}^{G,A}$ is not symmetric about the subsystems A and B.

## 3. Properties of M on Gaussian States

Let $M:\mathrm{FS}\left(H_{A}⊗H_{B}\right)\to \left[0,+\infty \right)$ be the function as Definition 1. M has several nice properties, whose proofs will be given in Appendix A.

**Theorem** **1.** *The following statements are true:*

(1) *M is independent of the mean of states;*
(2) *M is symmetric about the subsystems: for any state $\rho_{AB}\in \mathrm{FS}\left(H_{A}⊗H_{B}\right)$, $M\left(F\left(\rho_{AB}\right)\right)=M\left(\rho_{AB}\right)$, where $F:S\left(H_{A}⊗H_{B}\right)\to S\left(H_{B}⊗H_{A}\right)$ is the swap defined by $F\left(\rho_{A}⊗\rho_{B}\right)=\rho_{B}⊗\rho_{A}$.*
(3) *M has no ancilla problem: for any state $\rho_{C}\in \mathrm{FS}\left(H_{C}\right)$, regarding $\rho_{ABC}=\rho_{AB}⊗\rho_{C}$ as a bipartite state with partition A:BC, we always have $M\left(\rho_{ABC}\right)=M\left(\rho_{AB}\right)$.*

**Theorem** **2.** *M is locally Gaussian unitary invariant, that is, for any (n+m)-mode Gaussian state $\rho_{AB}\in S\left(H_{A}⊗H_{B}\right)$ and any Gaussian unitary operators $W\in B(H_{A})$ and $V\in B(H_{B})$, we have $M\left(\left(W⊗V\right)\rho_{AB}\left(W^{†}⊗V^{†}\right)\right)=M\left(\rho_{AB}\right)$.*

**Theorem** **3.** *For any (n+m)-mode state $\rho_{AB}\in S\left(H_{A}⊗H_{B}\right)$ with CM $\Gamma =\left(\begin{array}{cc} A & C\\ C^{T} & B \end{array}\right)$, $M(\rho_{AB})=0$ if and only if C=0. Particularly, for any Gaussian states $\rho_{AB}$, $M(\rho_{AB})=0$ if and only if $\rho_{AB}$ is a product state.*

By Theorem 3, for Gaussian states, M describes the same non-classicality as that described by Gaussian quantum discord (two-mode) [6,7], Gaussian geometric discord [8], the Gaussian discord of response $GD_{R}^{x}$ in [10], the correlations *Q*, $Q_{P}$ discussed in [12], the correlations $N_{F}$ and $N_{F}$ discussed, respectively, in [19,20], as they take value 0 at a Gaussian state $\rho_{AB}$ if and only if $\rho_{AB}$ is a product state.

According to Definition 1, M relies only on the CM of a given Gaussian state and is independent of the measurements and optimization process. Hence, unlike those Gaussian quantum correlations involved measurements, the estimate of M is easy and reliable. In the following, we are going to give some computation formulas of M based on the representations of CM of the Gaussian states.

For any (1+1)-mode Gaussian state $\rho_{AB}$, under some suitable local Gaussian unitary operation, its CM can be reduced to the standard form

(5) $$\Gamma_{0}=\left(\begin{array}{cc} A_{0} & C_{0}\\ C_{0}^{T} & B_{0} \end{array}\right)=\left(\begin{array}{cccc} a & 0 & c & 0\\ 0 & a & 0 & d\\ c & 0 & b & 0\\ 0 & d & 0 & b \end{array}\right).\;$$

Therefore, by Theorem 2, we have

**Theorem** **4.** *If $\rho_{AB}$ is a (1+1)-mode Gaussian state whose CM has the standard form Equation (5), then we have*

$$\begin{array}{c} M\left(\rho_{AB}\right)=1-\frac{\left(ab-c^{2}\right)\left(ab-d^{2}\right)}{a^{2}b^{2}}. \end{array}$$

Consider the (n+m)-mode pure Gaussian states. Without loss of generality, assume that n≤m. Then, according to the mode-wise decomposition of pure Gaussian states [30], the CM Γ of any (n+m)-mode pure Gaussian state can always be brought into $\Gamma_{S}$ by the corresponding symplectic transformation $S=S_{n}⊕S_{m}$. Moreover,

(6) $$\Gamma_{S}=S\Gamma S^{T}=\overset{n}{\underset{j=1}{⨁}}\left(\begin{array}{cccc} \gamma_{j} & 0 & \sqrt{\gamma_{j}^{2}-1} & 0\\ 0 & \gamma_{j} & 0 & -\sqrt{\gamma_{j}^{2}-1}\\ \sqrt{\gamma_{j}^{2}-1} & 0 & \gamma_{j} & 0\\ 0 & -\sqrt{\gamma_{j}^{2}-1} & 0 & \gamma_{j} \end{array}\right)⊕I_{2(m-n)}\;$$

with $\gamma_{j}\geq 1$, j=1,2,…,n, the single-mode mixedness factor.

The following results give computation formulas of M for, respectively, (n+m)-mode and (1+m)-mode pure Gaussian states in terms of the single-mode mixedness factor.

**Theorem** **5.** *Suppose n≤m, for any (n+m)-mode pure Gaussian state $\rho_{AB}$, let $\gamma_{j}\geq 1$, j=1,2,…,n, be the single-mode mixedness factors in the CM of the mode-wise decomposition of the pure Gaussian state. Then, we have*

$$M\left(\rho_{AB}\right)=1-\frac{1}{\prod_{j=1}^{n}\gamma_{j}^{4}}.$$

Particularly, any (1+m)-mode pure Gaussian state can always be brought in the phase-space Schmidt form [31]. The corresponding symplectic transformation S achieving the Schmidt decomposition is the direct sum of two diagonalizing matrices acting on the single-mode and *m*-mode subsystems, respectively, i.e., $S=S_{1}⊕S_{2}$. Suppose Γ is the CM of a (1+m)-mode pure Gaussian state; accordingly, the CM of its phase-space Schmidt form is

(7) $$\Gamma_{S}=S\Gamma S^{T}=\left(\begin{array}{cccc} \gamma & 0 & \sqrt{\gamma^{2}-1} & 0\\ 0 & \gamma & 0 & -\sqrt{\gamma^{2}-1}\\ \sqrt{\gamma^{2}-1} & 0 & \gamma & 0\\ 0 & -\sqrt{\gamma^{2}-1} & 0 & \gamma \end{array}\right)⊕I_{2(m-1)}\;$$

with γ≥1 the single-mode mixedness factor. We also call $\Gamma_{S}$ the phase-space Schmidt form of Γ. It is clear that the phase-space Schmidt form of a (1+m)-mode pure Gaussian state is the tensor product of a two-mode squeezed state and an (m−1)-mode uncorrelated vacuum state [32].

**Corollary** **1.** *For any (1+m)-mode pure Gaussian state $\rho_{AB}$, we have*

$$M\left(\rho_{AB}\right)=1-\frac{1}{\gamma^{4}},$$

*where γ≥1 is the single-mode mixedness factor in the phase-space Schmidt form of the CM Γ.*

The physical meaning of M is that $M\left(\rho_{AB}\right)>M\left(\sigma_{AB}\right)$ reveals that $\rho_{AB}$ is more correlated than $\sigma_{AB}$. To see this, let us consider the following example. According to the mode-wise decomposition of pure Gaussian states mentioned above, the phase-space Schmidt form of the CM of any (1+1)-mode pure Gaussian state $\rho_{AB}$ is

$$\Gamma_{S}=S\Gamma S^{T}=\left(\begin{array}{cccc} \gamma & 0 & \sqrt{\gamma^{2}-1} & 0\\ 0 & \gamma & 0 & -\sqrt{\gamma^{2}-1}\\ \sqrt{\gamma^{2}-1} & 0 & \gamma & 0\\ 0 & -\sqrt{\gamma^{2}-1} & 0 & \gamma \end{array}\right)≐\Gamma_{S}\left(\gamma \right),$$

where γ≥1 is the single-mode mixedness factor. In [33], a measure of entanglement D for (1+n)-mode pure Gaussian state is derived, where, for any (1+1)-mode pure Gaussian state $\rho_{AB}$ with $\Gamma_{S}\left(\gamma \right)$, the phase-space Schmidt form of the CM,

$$D\left(\rho_{AB}\right)=1-\frac{2}{\gamma^{2}+1}.$$

It is well known that for any entanglement measure *E*, and any states $\rho_{AB}$ and $\sigma_{AB}$, one may regard that $\rho_{AB}$ is more entangled than $\sigma_{AB}$ whenever $E\left(\rho_{AB}\right)>E\left(\sigma_{AB}\right)$. Then, for Gaussian pure state $\rho_{AB}$ with CM $\Gamma_{S}\left(\sqrt{3}\right)$ and $\sigma_{AB}$ with CM $\Gamma_{S}\left(\sqrt{2}\right)$, one has $D\left(\rho_{AB}\right)=1-\frac{2}{{\left(\sqrt{3}\right)}^{2}+1}=\frac{1}{2}$ and $D\left(\sigma_{AB}\right)=1-\frac{2}{{\left(\sqrt{2}\right)}^{2}+1}=\frac{1}{3}$, thus $D\left(\rho_{AB}\right)>D\left(\sigma_{AB}\right)$, i.e., $\rho_{AB}$ is more correlated than $\sigma_{AB}$. By Definition 1,

$$M\left(\rho_{AB}\right)=1-\frac{1}{\gamma^{4}}.$$

Therefore, $M\left(\rho_{AB}\right)=\frac{8}{9}>\frac{3}{4}=M\left(\sigma_{AB}\right)$, which reveals the same fact that $\rho_{AB}$ contains more correlation than $\sigma_{AB}$. Geometrically, $M\left(\rho_{AB}\right)>M\left(\sigma_{AB}\right)$ reflects that $\sigma_{AB}$ is closer to the set of product states than $\rho_{AB}$.

As mentioned before, M, *D*, $D_{G}$, *Q*, $N_{F}$, and $N_{F}$ describe the same non-classicality for (n+m)-mode Gaussian states. In [20], we compared the scales of $N_{F}^{G}$ with Gaussian quantum discord *D*, Gaussian Geometric Discord $D_{G}$ and quantum correlation *Q*, and found that, $N_{F}^{G}$ is the best one in detecting such non-locality. As an upper bound of $N_{F}^{G}$, M surely can do better.

To be specific, consider a special class of Gaussian states, the symmetric squeezed thermal states (SSTSs). Recall that the symmetric squeezed thermal states (SSTSs) are Gaussian states whose CMs are as in Equation (2), parameterized by $\bar{n}$ and μ such that $a=b=1+2\bar{n}$ and $c=-d=2\mu \sqrt{\bar{n}\left(1+\bar{n}\right)}$, where $\bar{n}$ is the mean photon number for each party and μ is the mixing parameter with 0≤μ≤1[34]. By Theorem 4, for any SSTS $\rho_{AB}$, we have

(8) $$M\left(\rho_{AB}\left(\bar{n},\mu \right)\right)=1-\frac{{\left({\left(1+2\bar{n}\right)}^{2}-4\mu^{2}\bar{n}\left(1+\bar{n}\right)\right)}^{2}}{{\left(1+2\bar{n}\right)}^{4}}.\;$$

According to the analytical formula provided in [8], for any SSTS $\rho_{AB}$ with parameters $\bar{n}$ and μ, one has -4.6cm0cm

(9) $$D_{G}\left(\rho_{AB}\left(\bar{n},\mu \right)\right)=\frac{1}{{\left(1+2\bar{n}\right)}^{2}-4\bar{n}\left(1+\bar{n}\right)\mu^{2}}-\frac{9}{{\left(\sqrt{{\left(1+2\bar{n}\right)}^{2}}+2\sqrt{{\left(1+2\bar{n}\right)}^{2}-3\bar{n}\left(1+\bar{n}\right)\mu^{2}}\right)}^{2}}.\;$$

**Figure 1:**
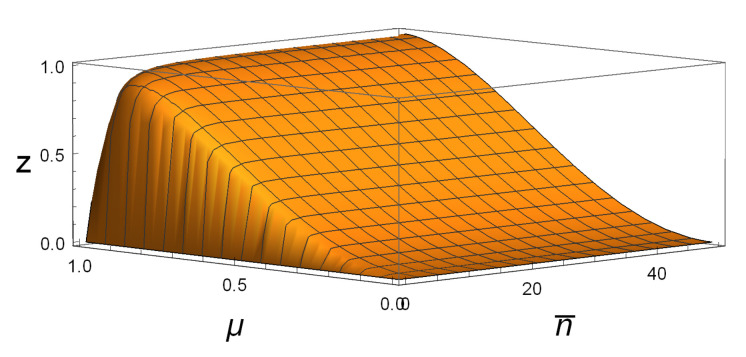
For SSTSs $\rho_{AB}\left(\bar{n},\mu \right)$ with 0≤μ≤1 and $0\leq \bar{n}\leq 50$, z=$M\left(\rho_{AB}\left(\bar{n},\mu \right)\right)-D_{G}\left(\rho_{AB}\left(\bar{n},\mu \right)\right)$, it is clear that the figure is above the $\bar{n}o\mu$ plane.

Figure 1 shows that $M\left(\rho_{AB}\left(\bar{n},\mu \right)\right)>D_{G}\left(\rho_{AB}\left(\bar{n},\mu \right)\right)$ for all SSTSs with 0<μ≤1 and $0\leq \bar{n}\leq 50$. For example, taking $\bar{n}=40$ and μ=0.8, one sees that $D_{G}\left(\rho_{AB}\left(40,0.8\right)\right)\approx 0.00019\approx 0$, while $M(\rho_{AB}\left(40,0.8\right))\approx 0.87033\gg 0$. This suggests that $M(\rho_{AB})$ is better in detecting whether or not a state is a product state.

## 4. Non-Increasing Property of M under Local Gaussian Operations

As a Gaussian state ρ is described by its CM Γ and displacement vector d, we can denote it as ρ=ρ(Γ,d). Recall that a Gaussian channel is a quantum channel that transforms Gaussian states into Gaussian states. Assume that Φ is a Gaussian channel of *n*-mode Gaussian systems. Then, there exist real matrices $M,K\in M_{2n}\left(R\right)$ satisfying $M=M^{T}\geq 0$ and det $M\geq {\left(\det K-1\right)}^{2}$, and a vector $\bar{d}\in R^{2n}$, such that, for any *n*-mode Gaussian state ρ=ρ(Γ,d), we have $\Phi \left(\rho \left(\Gamma ,d\right)\right)=\rho \left(\Gamma^{\prime },d^{\prime }\right)$ with

(10) $$d^{\prime }=Kd+\bar{d}\;\;\mathrm{and}\;\;\Gamma^{\prime }=K\Gamma K^{T}+M.\;$$

Therefore, we can parameterize the Gaussian channel Φ as $\Phi =\Phi (K,M,\bar{d})$.

We first consider the (1+1)-mode Gaussian states. As M is invariant under local Gaussian unitary operation, we may require that the CM of involved Gaussian state is of the standard form.

**Theorem** **6.** *Consider the (1+1)-mode continuous-variable system AB. Let $\Phi =\Phi (K,M,\bar{d})$ be a Gaussian channel performed on the subsystem B with $K=\left(\begin{array}{cc} k_{11} & k_{12}\\ k_{21} & k_{22} \end{array}\right)$ and $M=\left(\begin{array}{cc} m_{11} & m_{12}\\ m_{12} & m_{22} \end{array}\right)$. Assume that $\rho_{AB}\in S\left(H_{A}⊗H_{B}\right)$ is any (1+1)-mode Gaussian state with CM $\Gamma_{0}=\left(\begin{array}{cccc} a & 0 & c & 0\\ 0 & a & 0 & d\\ c & 0 & b & 0\\ 0 & d & 0 & b \end{array}\right)$. Then,*

$$\begin{array}{c} M\left(\left(I⊗\Phi \right)\rho_{AB}\right)=1-\frac{\left(ab-c^{2}\right)\left(ab-d^{2}\right)n_{1}+a\left(ab-c^{2}\right)n_{2}+a\left(ab-d^{2}\right)n_{3}+a^{2}n_{4}}{a^{2}b^{2}n_{1}+a^{2}b\left(n_{2}+n_{3}\right)+a^{2}n_{4}}, \end{array}$$

*where $n_{1}=k_{11}^{2}k_{22}^{2}+k_{12}^{2}k_{21}^{2}-2k_{11}k_{12}k_{21}k_{22}$, $n_{2}=m_{22}k_{11}^{2}+m_{11}k_{21}^{2}-2m_{12}k_{11}k_{21}$, $n_{3}=m_{22}k_{12}^{2}+m_{11}k_{22}^{2}-2m_{12}k_{12}k_{22}$ and $n_{4}=m_{11}m_{22}-m_{12}^{2}$.*

**Remark** **1.** *If K=0, then detM≥1, and we have*

$$\begin{array}{cc} M(\left(I⊗\Phi \right)\rho_{AB})= & 1-\frac{\det M}{\det M}=0. \end{array}$$

*In fact, in this case, the Gaussian channel $I⊗\Phi (0,M,\bar{d})$ maps any Gaussian state $\rho_{AB}$ to a product state. Thus, by Theorem 3, we always have $M(\left(I⊗\Phi \right)\rho_{AB})=0$.*

**Remark** **2.** *If M=0, then $\det K=1=\det K^{T}$, and*

$$\begin{array}{cc} M(\left(I⊗\Phi \right)\rho_{AB})= & 1-\frac{\det(K\left(B_{0}-C_{0}^{T}A_{0}^{-1}C_{0}\right)K^{T})}{\det(KB_{0}K^{T})}\\ = & 1-\frac{\det(B_{0}-C_{0}^{T}A_{0}^{-1}C_{0})}{\det B_{0}}\\ = & M(\rho_{AB}). \end{array}$$

*Thus, in this case, after performing the Gaussian operation $I⊗\Phi (K,0,\bar{d})$, the quantity M remains the same.*

As a consequence of Theorem 6, the following result gives a stronger form of *local Gaussian operation non-increasing property* of M, which is not possessed by other known similar Gaussian correlations such as the Gaussian quantum discord (two-mode) [6,7], Gaussian geometric discord [8], and the Gaussian quantum correlation $N_{F}$ in [19].

**Corollary** **2.** *Let $\rho_{AB}$ be a (1+1)-mode Gaussian state. Then, for any Gaussian channels $\Phi_{A}$ and $\Phi_{B}$ performed on the subsystem A and B, respectively, we have*

$$\begin{array}{c} 0\leq M\left(\left(\Phi_{A}⊗\Phi_{B}\right)\rho_{AB}\right)\leq M\left(\rho_{AB}\right). \end{array}$$

It is remarkable that the result of Corollary 2 is true for any (m+n)-mode systems; that is, we have the following.

**Theorem** **7.** *For any (m+n)-mode Gaussian state $\rho_{AB}$, for any Gaussian channels $\Phi_{A}$ and $\Phi_{B}$ performed on the subsystem A and B respectively, we have*

$$\begin{array}{c} 0\leq M\left(\left(\Phi_{A}⊗\Phi_{B}\right)\rho_{AB}\right)\leq M\left(\rho_{AB}\right). \end{array}$$

Obviously, Theorem 7 implies Theorem 2, the local Gaussian unitary invariance.

Theorem 7, together with Theorems 1–3, reveal that M is a Gaussian quantum correlation without ancilla problem which describes the same Gaussian quantum correlation as the Gaussian quantum discord and the Gaussian geometric discord for (m+n)-mode Gaussian systems. An (n+m)-mode Gaussian state has this correlation if and only if it is not a product state. We remark here that, just like the entanglement, the non-product correlation is symmetric about the subsystems. Therefore, it is more natural to require that a non-product correlation measure is symmetric about the subsystems. Our M has this symmetry, but all known such Gaussian correlations are introduced by some local operations on a subsystem and thus not symmetric about the subsystem.

## 5. A Possible Future Application of M: Thermometry

Intracellular temperature measurement is a key point in the field of life science, and scientists have invented nanothermometers for detecting intracellular temperatures. Uchiyama detected and depicted the temperature distribution of a single cell by implanting special nanogels into the cytoplasm [35]. Other methods for measuring intracellular temperature can be found in [36,37], and those nanothermometers detect intracellular temperature by sending special luminescent or polymer materials into the cell. As the Gaussian correlation M is computable for any (n+m)-mode Gaussian states, it is easier to be applied in real-life scenarios such as the quantum information tasks and quantum biology scenarios. In this section, we give a possible application of M to thermometry, which is currently on theoretical level. In the following, we briefly describe a possible quantum method of measuring intracellular temperature by the Gaussian quantum correlation M.

To implement the quantum method, first, one prepare laser beam (Gaussian state) $\rho_{G}\left(0\right)$ with quantum correlation as the initial state, and put this Gaussian state into a specific cell in certain tissue or organ by laser irradiation. The laser beam is so small that we can consider the cell as the environment system of the Gaussian state. The cell and the Gaussian state constitute a composite system, ignoring the effect of extracellular environment, the composite system can be treated approximately as a closed system. Obviously, the Gaussian state is not related to the environment the moment it enters the cell, as a consequence, the initial state of the composite system can written as $\rho_{G}\left(0\right)⊗\sigma \left(0\right)$, where σ(0) stands for the cell sate. As the cell has temperature, we can view the cell as a thermal environment of the Gaussian state. Thus, the Gaussian state follows the thermodynamic evolution law related to environment temperature *T* in the cell. During the evolution process, the environment will affect the Gaussian state $\rho_{G}\left(0\right)$ in the subsystem (we only consider the affect of intracellular temperature *T* here). Detecting the quantities of quantum correlation M contained in the evolved state $\rho_{G}\left(t,T\right)$ at time *t* by proper detector, after calculation, one gets the intracellular temperature of the cell.

As an illustration, and for simplicity’s sake, assume that the system which prepares the initial Gaussian state is a (1+1)-mode boson system $H_{G}$, denote $m_{k}$ and $w_{k}$ as the mass and frequency of the *k*-th resonator, let ${\hat{Q}}_{k}$ and ${\hat{P}}_{k}$ stand for the momentum and position operator of the *k*-th mode, where k=1,2. Let $H_{E}$ represent the thermal environment system (the cell), then the composite system coupled by the Gaussian state and the cell is $H_{G}⊗H_{E}$. Apparently, the product state $\rho_{GE}\left(0\right)=\rho_{G}\left(0\right)⊗\rho_{E}\left(0\right)$ is the initial state of the coupled composite system, where $\rho_{G}\left(0\right)\in B\left(H_{G}\right)$ is a Gaussian state with mean m(0) and covariance matrix Γ(0), and $\rho_{E}\left(0\right)\in B\left(H_{E}\right)$ stands for the cell state. Consider approximatively the composite system as a closed system, then the time evolution of the initial Gaussian state $\rho_{GE}\left(0\right)$ is unitary: $\rho_{GE}\left(t\right)=U_{GE}\left(t\right)\rho_{GE}\left(0\right)U_{GE}^{†}\left(t\right)$, where the evolution operator $U_{GE}$ depends on the Hamiltonian of the composite system. However, affected by the environment system, the time evolution of the reduced Gaussian state $\rho_{G}\left(0\right)$, $\rho_{G}\left(t\right)={\mathrm{Tr}}_{E}\left(U_{GE}\left(t\right)\rho_{GE}\left(0\right)U_{GE}^{†}\left(t\right)\right)$ is no longer unitary evolution of $\rho_{G}\left(0\right)$, instead, it is determined by a time-dependent Gaussian channel.

As M is locally unitary invariant and independent of the mean, without loss of generality, we can prepare (1+1)-mode squeezed thermal state $\rho_{G}\left(0\right)$ as the initial state, with covariance matrix

$$\Gamma \left(0\right)=\left(\begin{array}{cccc} a & 0 & c & 0\\ 0 & a & 0 & -c\\ c & 0 & b & 0\\ 0 & -c & 0 & b \end{array}\right),$$

where a,b related to the compression parameters and the average photon number per mode, while *c* depends on the compression parameters and the average photon number on two modes. Let a=b=2, c=1, $m_{1}=m_{2}=\omega_{1}=\omega_{2}=\hbar =1$. Then, by an approach as in [38,39], one gets the covariance matrix Γ(t) of the time revolution $\rho_{G}\left(t\right)$ is

(11) $$\Gamma \left(t\right)=\left(\begin{array}{cccc} m(t,T) & 0 & 1 & 0\\ 0 & m(t,T) & 0 & -1\\ 1 & 0 & m(t,T) & 0\\ 0 & -1 & 0 & m(t,T) \end{array}\right),\;$$

where $m\left(t,T\right)=e^{-2t}\left(2-\frac{1}{2}\coth\frac{1}{2KT}\right)+\frac{1}{2}\coth\frac{1}{2KT}$, with *K* the Boltzmann constant. A proof of Equation (11) will be given in Appendix A.

Now, by Theorem 4, at time *t*, and under the influence of cell environment *T*, the quantity of quantum correlation $M(\rho_{G}\left(t\right))$ of the Gaussian state $\rho_{G}\left(t\right)$ is

(12) $$M\left(\rho_{G}\left(t\right)\right)=1-\frac{{\left({\left(e^{-2t}\left(2-\frac{1}{2}\coth\frac{1}{2KT}\right)+\frac{1}{2}\coth\frac{1}{2KT}\right)}^{2}-1\right)}^{2}}{{\left(e^{-2t}\left(2-\frac{1}{2}\coth\frac{1}{2KT}\right)+\frac{1}{2}\coth\frac{1}{2KT}\right)}^{4}},\;$$

which depends on time *t* and the intracellular temperature *T*. Hence, we may write $\rho_{G}\left(t\right)$ as $\rho_{G}\left(t,T\right)$. Thus, once we measured the quantity $M(\rho_{G}\left(t,T\right))$ of the Gaussian state $\rho_{G}\left(t,T\right)$ at time *t*, the intracellular temperature *T* can be easily drawn from the Equation (12), i.e., the intracellular temperature of the cell is detected.

We point out that, in our model, one may choose different detectors which can detect other quantum correlations contained in Gaussian state $\rho_{G}\left(t,T\right)$, while among which, the computation of M is so far the simplest one.

In the following, under the settings of the above model, we investigate the change trend of quantum correlation $M(\rho_{G}\left(t,T\right))$.

Let $\alpha =\coth\frac{1}{2KT}$; it is clear that α is a monotone increasing function of Intracellular temperature *T*, and one can write Equation (12) as

(13) $$M\left(\rho_{G}\left(t,\alpha \right)\right)=1-\frac{{\left({\left(e^{-2t}\left(2-\frac{1}{2}\alpha \right)+\frac{1}{2}\alpha \right)}^{2}-1\right)}^{2}}{{\left(e^{-2t}\left(2-\frac{1}{2}\alpha \right)+\frac{1}{2}\alpha \right)}^{4}}.\;$$

**Figure 2:**
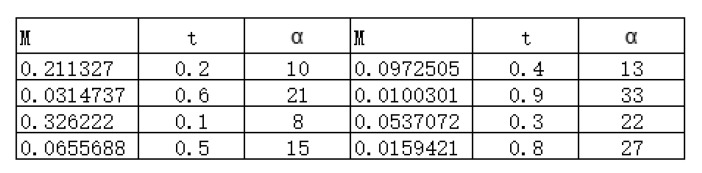
In the cellular environment, the relation between $M(\rho_{G}\left(t,\alpha \right))$, t and α.

Figure 2 shows the corresponding relation between $M(\rho_{G}\left(t,\alpha \right))$, *t*, and α. Apparently, once the quantity $M(\rho_{G}\left(t,\alpha \right))$ of the Gaussian state $\rho_{G}\left(t,\alpha \right)$ at time *t* is detected, one can solve α by Equation (13), and further, the intracellular temperature *T*.

**Figure 3:**
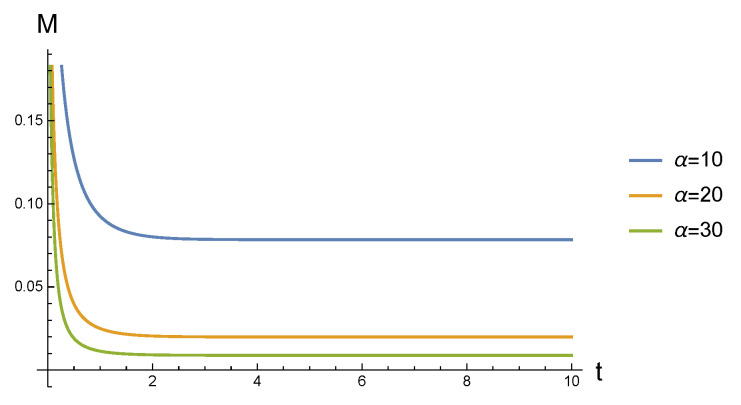
In cell environment, for different α, the dynamic evolution properties of $M(\rho_{G}\left(t,\alpha \right))$.

Fix α=10, α=20, and α=30, in Figure 3, we delineate the evolution behaviors of quantum correlation $M(\rho_{G}\left(t,\alpha \right))$ the cellular environment with blue, orange, and green curves, respectively. Clearly, $M(\rho_{G}\left(t,\alpha \right))$ decrease dramatically in a short time at the beginning, after that, it becomes stable. Figure 2 also reveals that, as α gets bigger(the temperature gets higher), the speed and the amplitude of the attenuation of $M(\rho_{G}\left(t,\alpha \right))$ gets greater, i.e., when α is small, it takes more time for $M(\rho_{G}\left(t,\alpha \right))$ to become stable. This means that when a Gaussian state is coupled with the cellular environment, the revolution of quantum correlation M contained in Gaussian state is a feedback of the intracellular temperature. To be specific, the greater the speed and amplitude of the attenuation of M, the higher the intracellular temperature.

## 6. Conclusions

By now, all quantifications of Gaussian quantum discord and Gaussian geometric discord for (n+m)-mode bipartite continuous-variable systems have been derived from considering the difference between the Gaussian state and the output after performing some measurements over certain subsystem, and then, taking an optimization procedure. The obstacle for applying these quantifications of Gaussian quantum discord is that they are very difficult to be calculated, though a lot of effort have be paid.

The main work of the present paper is to propose a new quantification M in terms of covariant matrices for any states in (n+m)-mode continuous-variable systems without any measurements performed on a subsystem and any optimization procedures. This quantification M has many attractive properties: M is independent of the mean of states, is symmetric about the subsystems, has no ancilla problem, and is easily computed for any (n+m)-mode Gaussian states. M is locally Gaussian unitary invariant and is increasing under local Gaussian channels, that is, $0\leq M\left(\left(\Phi_{A}⊗\Phi_{B}\right)\rho_{AB}\right)\leq M\left(\rho_{AB}\right)$ holds for any Gaussian channels $\Phi_{A}$ and $\Phi_{B}$ performed on the subsystem A and B, respectively. $M(\rho_{AB})=0$ if and only if $\rho_{AB}$ is a product state. M is an upper bound of a replacement of Gaussian geometric discord, $N_{F}$, which is defined and discussed in [20]. Therefore, M is a Gaussian correlation which is a very nice replacement of Gaussian quantum discord as well as Gaussian geometric discord. As an application of M, a noninvasive quantum method for detecting intracellular temperature is proposed.

We remark that, unlike the other known Gaussian quantum correlations, M is symmetric about the subsystem A and B. Thus, as a Gaussian quantum correlation, M is more natural because the property that a state is not a product state is symmetric about the subsystems. Moreover, the concepts of Gaussian quantum discord and Gaussian geometric discord are very difficult to extend to multipartite multimode continuous-variable systems, however, the definition of M can be generalized naturally to any states for multipartite multimode continuous-variable systems. This gives some possibility to discuss the problem of quantifying the Gaussian quantum correlation in multipartite multimode continuous-variable systems.

## Appendix A

In this appendix, we present our proofs of Theorems 1–7.

**Proof** **of** **Theorem** **1.** The properties that M is independent of the mean and is symmetric about the subsystems are obvious from Definition 1. Assume that $\rho_{AB}\in \mathrm{FS}\left(H_{A}⊗H_{B}\right)$ is any (n+m)-mode Gaussian state with CM $\Gamma =\left(\begin{array}{cc} A & C\\ C^{T} & B \end{array}\right)$. Then, the corresponding CM of the swapped state $F\left(\rho_{AB}\right)\in \mathrm{FS}\left(H_{B}⊗H_{A}\right)$ is $\tilde{\Gamma }=\left(\begin{array}{cc} B & C^{T}\\ C & A \end{array}\right)$, by Definition 1, one has $M\left(F\left(\rho_{AB}\right)\right)=M\left(\rho_{AB}\right)$. To see that M has no ancilla problem, when an uncorrelated ancilla system C is appended, the corresponding CM of $\rho_{A:BC}$ has the form of $\bar{\Gamma }=\left(\begin{array}{ccc} A & C & 0\\ C^{T} & B & 0\\ 0 & 0 & D \end{array}\right)$. It follows that

$$\begin{array}{cc} M(\rho_{A:BC})= & 1-\frac{\det(\left(\begin{array}{cc} B & 0\\ 0 & D \end{array}\right)-\left(\begin{array}{c} C^{T}\\ 0 \end{array}\right)A^{-1}\left(C\;\;\;0\right))}{\det\left(\begin{array}{cc} B & 0\\ 0 & D \end{array}\right)}\\ = & 1-\frac{\det\left(\begin{array}{cc} B-C^{T}A^{-1}C & 0\\ 0 & D \end{array}\right)}{\det B\det D}\\ = & 1-\frac{\det(B-C^{T}A^{-1}C)}{\det B}\\ = & M(\rho_{AB}), \end{array}$$

completing the proof. □

**Proof** **of** **Theorem** **2.** Assume that $\rho_{AB}\in S\left(H_{A}⊗H_{B}\right)$ is any (n+m)-mode Gaussian state with CM $\Gamma =\left(\begin{array}{cc} A & C\\ C^{T} & B \end{array}\right)$. For given Gaussian unitary operators $W\in B(H_{A})$ and $V\in B(H_{B})$, let $\sigma_{AB}=\left(W⊗V\right)\rho_{AB}\left(W^{†}⊗V^{†}\right)$. According to the Williamson Theorem, a Gaussian unitary operator corresponding to a symplectic matrix in the CM level. Denote by $S_{W}$ and $S_{V}$, respectively, the corresponding symplectic matrixes. Then, the CM of $\sigma_{AB}$ is

$$\begin{array}{cc} & \bar{\Gamma }=\left(\begin{array}{cc} S_{W} & 0\\ 0 & S_{V} \end{array}\right)\left(\begin{array}{cc} A & C\\ C^{T} & B \end{array}\right)\left(\begin{array}{cc} S_{W}^{T} & 0\\ 0 & S_{V}^{T} \end{array}\right)=\left(\begin{array}{cc} S_{W}AS_{W}^{T} & S_{W}CS_{V}^{T}\\ S_{V}C^{T}S_{W}^{T} & S_{V}BS_{V}^{T} \end{array}\right). \end{array}$$

By definition,

$$\begin{array}{cc} M(\left(W⊗V\right)\rho_{AB}\left(W^{†}⊗V^{†}\right))= & 1-\frac{\det(S_{V}BS_{V}^{T}-S_{V}C^{T}S_{W}^{T}{\left(S_{W}AS_{W}^{T}\right)}^{-1}S_{W}CS_{V}^{T})}{\det(S_{V}BS_{V}^{T})}\\ = & 1-\frac{\det(S_{V}BS_{V}^{T}-S_{V}C^{T}A^{-1}CS_{V}^{T})}{\det(S_{V}BS_{V}^{T})}\\ = & 1-\frac{\det S_{V}\det\left(B-C^{T}A^{-1}C\right)\det S_{V}^{T}}{\det S_{V}\det B\det S_{V}^{T}}\\ = & 1-\frac{\det(B-C^{T}A^{-1}C)}{\det B}\\ = & M(\rho_{AB}) \end{array}$$

as desired. The proof completes. □

To prove Theorem 3, we need the following Lemma.

**Lemma** **A1.**
*Let $A,B\in M_{n}\left(C\right)$, then A≥B≥0 with B is invertible. If detA=detB, then A=B.*

**Proof** **of** **Lemma** **A1.** As *B* is invertible, then detA=detB>0. Assume that, for k=1,2,…,n, $\lambda_{k}^{A}$ and $\lambda_{k}^{B}$ are, respectively, eigenvalues of *A* and *B*, and they are arranged in descending order, i.e., $\lambda_{1}^{A}\geq \lambda_{2}^{A}\geq \cdot \cdot \cdot \geq \lambda_{n}^{A}>0$, $\lambda_{1}^{B}\geq \lambda_{2}^{B}\geq \cdot \cdot \cdot \geq \lambda_{n}^{B}>0$. As A≥B≥0, according to the corollary 7.7.4 in [29], one has TrA≥TrB and $\lambda_{k}^{A}\geq \lambda_{k}^{B}$ for k=1,2,…,n. If detB=detA, then $\prod_{k=1}^{n}\lambda_{k}^{B}=\prod_{k=1}^{n}\lambda_{k}^{A}$, which forces $\lambda_{k}^{A}=\lambda_{k}^{B}$ for k=1,2,…,n. Consequently, we have $\mathrm{Tr}B=\sum_{k=1}^{n}\lambda_{k}^{B}=\sum_{k=1}^{n}\lambda_{k}^{A}=\mathrm{Tr}A$. Without loss of generality, suppose $B={\left(b_{kl}\right)}_{n\times n}$ and $A=\mathrm{diag}(\lambda_{1}^{A},\lambda_{2}^{A},\cdot \cdot \cdot ,\lambda_{n}^{A})$. Now, A−B≥0 implies that $\lambda_{k}^{A}-b_{kk}\geq 0$. As $\sum_{k=1}^{n}b_{kk}=\mathrm{Tr}B=\mathrm{Tr}A=\sum_{k=1}^{n}\lambda_{k}^{A}$, we must have $\lambda_{k}^{A}=b_{kk}$ for each k=1,2,…,n, which entails that $b_{kl}=0$ whenever k≠l since A−B≥0. Therefore, one gets A=B, as desired. □

**Proof** **of** **Theorem** **3.** Assume that $\rho_{AB}\in \mathrm{FS}\left(H_{A}⊗H_{B}\right)$ is an (n+m)-mode state with CM $\Gamma =\left(\begin{array}{cc} A & C\\ C^{T} & B \end{array}\right)$ as in Equation (1). If C=0, by Definition 1, it is obvious that $M\left(\rho_{AB}\right)=1-\frac{\det A\det B}{\det A\det B}=0$. Conversely, assume that $M(\rho_{AB})=0$, according to Definition 1, one must have $\det(B-C^{T}A^{-1}C)=\det B$. Let $D=B-C^{T}A^{-1}C$. It is clear that 0≤D≤B. Thus, by Lemma 1, we must have B=D, which follows that C=0. The last assertion is true because, for a bipartite Gaussian state $\rho_{AB}$, C=0 if and only if $\rho_{AB}$ is a product state. □

**Proof** **of** **Theorem** **5.** By Theorem 2, M is locally Gaussian unitary invariant. Therefore, for any (n+m)-mode pure Gaussian state $\rho_{AB}$, it is sufficient to assume that the CM Γ has the form as Equation (6). In this case, one sees that

$$A=\left(\begin{array}{cccc} \alpha_{1} & 0 & \ldots & 0\\ 0 & \alpha_{2} & \ldots & 0\\ \vdots & \vdots & \ddots & \vdots \\ 0 & 0 & \ldots & \alpha_{n} \end{array}\right),B=\left(\begin{array}{cccc} \beta_{1} & 0 & \ldots & 0\\ 0 & \beta_{2} & \ldots & 0\\ \vdots & \vdots & \ddots & \vdots \\ 0 & 0 & \ldots & \beta_{m} \end{array}\right)⊕I_{2(m-n)},C=\left(\begin{array}{ccccccc} \varepsilon_{1} & 0 & \ldots & 0 & 0 & \ldots & 0\\ 0 & \varepsilon_{2} & \ldots & 0 & 0 & \ldots & 0\\ \vdots & \vdots & \ddots & \vdots & \vdots & \ddots & \vdots \\ 0 & 0 & \ldots & \varepsilon_{n} & 0 & \ldots & 0 \end{array}\right),$$

where $\alpha_{j}=\beta_{j}=\left(\begin{array}{cc} \gamma_{j} & 0\\ 0 & \gamma_{j} \end{array}\right)$ and $\varepsilon_{j}=\left(\begin{array}{cc} \sqrt{\gamma_{j}^{2}-1} & 0\\ 0 & -\sqrt{\gamma_{j}^{2}-1} \end{array}\right)$ with $\gamma_{j}$ the *j*-th single-mode mixedness factor. Then, it is easy to check that

$$B-C^{T}A^{-1}C=\left(\begin{array}{cccc} \beta_{1}-\varepsilon_{1}\alpha_{1}^{-1}\varepsilon_{1} & 0 & \ldots & 0\\ 0 & \beta_{2}-\varepsilon_{2}\alpha_{2}^{-1}\varepsilon_{2} & \ldots & 0\\ \vdots & \vdots & \ddots & \vdots \\ 0 & 0 & \ldots & 0\\ 0 & 0 & \ldots & \beta_{n}-\varepsilon_{n}\alpha_{n}^{-1}\varepsilon_{n} \end{array}\right)⊕I_{2(m-n)}.$$

After some straightforward calculations, one gets

$$\det B=\prod_{j=1}^{n}\det\beta_{j}=\prod_{j=1}^{n}\gamma_{j}^{2},$$

$$\det\left(B-C^{T}A^{-1}C\right)=\prod_{j=1}^{n}\det\left(\beta_{j}-\varepsilon_{j}\alpha_{j}^{-1}\varepsilon_{j}\right)=\frac{1}{\prod_{j=1}^{n}\gamma_{j}^{2}}.$$

Therefore, $M\left(\rho_{AB}\right)=1-\frac{\det(B-C^{T}A^{-1}C)}{\det B}=1-\frac{1}{\prod_{j=1}^{n}\gamma_{j}^{4}}$. □

**Proof** **of** **Theorem** **6.** Suppose that the (1+1)-mode Gaussian state $\rho_{AB}$ has CM $\Gamma_{0}=\left(\begin{array}{cccc} a & 0 & c & 0\\ 0 & a & 0 & d\\ c & 0 & b & 0\\ 0 & d & 0 & b \end{array}\right)$. Then, by Equation (10), the CM $\Gamma^{\prime }$ of $\sigma_{AB}=\left(I⊗\Phi \right)\rho_{AB}$ is

$$\begin{array}{cc} \; & \Gamma^{\prime }=\left(\begin{array}{cc} I & 0\\ 0 & K \end{array}\right)\left(\begin{array}{cc} A_{0} & C_{0}\\ C_{0}^{T} & B_{0} \end{array}\right)\left(\begin{array}{cc} I & 0\\ 0 & K^{T} \end{array}\right)+\left(\begin{array}{cc} 0 & 0\\ 0 & M \end{array}\right)=\left(\begin{array}{cc} A_{0} & C_{0}K^{T}\\ KC_{0}^{T} & KB_{0}K^{T}+M \end{array}\right). \end{array}$$

After some straightforward calculations, one can immediately achieve that

$$\begin{array}{cc} \; & M\left(\left(I⊗\Phi \right)\rho_{AB}\right)=M\left(\sigma_{AB}\right)\\ = & 1-\frac{\det(\left(KB_{0}K^{T}+M\right)-KC_{0}^{T}A_{0}^{-1}C_{0}K^{T})}{\det(KB_{0}K^{T}+M)}. \end{array}$$

Clearly, *K*, *M* can not be zero simultaneously. After some tedious calculations, one gets

$$\begin{array}{ccc} & & M(\left(I⊗\Phi \right)\rho_{AB})\\ & = & 1-\frac{\left(ab-c^{2}\right)\left(ab-d^{2}\right)n_{1}+a\left(ab-c^{2}\right)n_{2}+a\left(ab-d^{2}\right)n_{3}+a^{2}n_{4}}{a^{2}b^{2}n_{1}+a^{2}b\left(n_{2}+n_{3}\right)+a^{2}n_{4}}, \end{array}$$

where

$$\begin{array}{cccc} n_{1}= & k_{11}^{2}k_{22}^{2}+k_{12}^{2}k_{21}^{2}-2k_{11}k_{12}k_{21}k_{22},\; & n_{2}= & m_{22}k_{11}^{2}+m_{11}k_{21}^{2}-2m_{12}k_{11}k_{21},\\ n_{3}= & m_{22}k_{12}^{2}+m_{11}k_{22}^{2}-2m_{12}k_{12}k_{22}, & n_{4}= & m_{11}m_{22}-m_{12}^{2}. \end{array}$$

The proof is completed. □

**Proof** **of** **Corollary** **2.** We first consider the special case that $\Phi_{A}=I$, and will prove that

(A1) $$0\leq M\left(\left(I⊗\Phi_{B}\right)\rho_{AB}\right)\leq M\left(\rho_{AB}\right).$$

To this end, assume that the (1+1)-mode Gaussian state $\rho_{AB}$ has CM $\Gamma_{0}$ of the standard form, that is, $\Gamma_{0}=\left(\begin{array}{cccc} a & 0 & c & 0\\ 0 & a & 0 & d\\ c & 0 & b & 0\\ 0 & d & 0 & b \end{array}\right).$ Let $\Phi_{B}=\Phi_{B}\left(K,M,\bar{d}\right)$ be any Gaussian channel performed on the subsystem B with $K=\left(\begin{array}{cc} k_{11} & k_{12}\\ k_{21} & k_{22} \end{array}\right)$ and $M=\left(\begin{array}{cc} m_{11} & m_{12}\\ m_{12} & m_{22} \end{array}\right).$ We have to show that $M\left(\left(I⊗\Phi \right)\rho_{AB}\right)\leq M\left(\rho_{AB}\right)$. If $M(\rho_{AB})=0$, then, by Theorem 3, $\rho_{AB}$ is a product state. Therefore, $\left(I⊗\Phi \right)\rho_{AB}$ is a product state, and thus $M\left(\left(I⊗\Phi \right)\rho_{AB}\right)=0=M\left(\rho_{AB}\right)$. Assume that $M(\rho_{AB})\neq 0$. Then, $M\left(\left(I⊗\Phi \right)\rho_{AB}\right)\leq M\left(\rho_{AB}\right)$ holds if and only if $\frac{M(\left(I⊗\Phi \right)\rho_{AB})}{M(\rho_{AB})}\leq 1$. Let $\alpha =\left(ab-c^{2}\right)\left(ab-d^{2}\right)$, $\beta =a^{2}b^{2}$, $\gamma =a\left(ab-c^{2}\right)n_{2}+a\left(ab-d^{2}\right)n_{3}+a^{2}n_{4}$ and $\delta =a^{2}b\left(n_{2}+n_{3}\right)+a^{2}n_{4}$ with $n_{2},n_{3},n_{4}$ as in Theorem 6. Then, according to Theorem 6, we have

$$\frac{M(\left(I⊗\Phi \right)\rho_{AB})}{M(\rho_{AB})}\leq 1\Leftrightarrow \frac{1-\frac{\alpha n_{1}+\gamma }{\beta n_{1}+\delta }}{1-\frac{\alpha }{\beta }}\leq 1\Leftrightarrow \frac{\alpha n_{1}+\gamma }{\beta n_{1}+\delta }\geq \frac{\alpha }{\beta }\Leftrightarrow \gamma \beta \geq \alpha \delta .$$

Therefore, it suffices to prove that γβ−αδ≥0. By some computations, one sees that

$$\begin{array}{ccc} & & \gamma \beta =\left[a\left(ab-c^{2}\right)n_{2}+a\left(ab-d^{2}\right)n_{3}+a^{2}n_{4}\right]a^{2}b^{2}\\ & = & a^{3}b^{2}\left(ab-c^{2}\right)n_{2}+a^{3}b^{2}\left(ab-d^{2}\right)n_{3}+a^{4}b^{2}n_{4} \end{array}$$

and

$$\begin{array}{ccc} & & \alpha \delta =a^{2}b\left(ab-c^{2}\right)\left(ab-d^{2}\right)n_{2}+a^{2}b\left(ab-c^{2}\right)\left(ab-d^{2}\right)n_{3}+a^{2}\left(ab-c^{2}\right)\left(ab-d^{2}\right)n_{4}. \end{array}$$

Note that $n_{1}=k_{11}^{2}k_{22}^{2}+k_{12}^{2}k_{21}^{2}-2k_{11}k_{12}k_{21}k_{22}={\left(k_{11}k_{22}-k_{12}k_{21}\right)}^{2}\geq 0$ and $n_{4}=m_{11}m_{22}-m_{12}^{2}=\det M\geq 0$. As $m_{22}k_{11}^{2}+m_{11}k_{21}^{2}\geq 2{\sqrt{m}}_{22}{\sqrt{m}}_{11}k_{11}k_{21}\geq 2m_{12}k_{11}k_{21}$, we have $n_{2}\geq 0$. One can verify $n_{3}\geq 0$ by the same way. Also note that a,b≥1 and $ab\geq c^{2}\left(d^{2}\right)$ by the constraint condition of the parameters in the definition of the Gaussian state. Now it is clear that

$$\begin{array}{ccc} & & \gamma \beta -\alpha \delta =a^{2}bd^{2}\left(ab-c^{2}\right)n_{2}+a^{2}bc^{2}\left(ab-d^{2}\right)n_{3}+a^{2}\left(abc^{2}+abd^{2}-c^{2}d^{2}\right)n_{4}\geq 0, \end{array}$$

as desired. To this end, we come to the conclusion that $M\left(\left(I⊗\Phi \right)\rho_{AB}\right)\leq M\left(\rho_{AB}\right)$, and the equality holds if M=0 (See Remark 2 after the proof of Theorem 6). Now let us consider the general case. Let U⊗V be a local Gaussian unitary operation, that is, for some Gaussian unitary operators *U* and *V* on the subsystems A and B, respectively, so that $\left(U⊗V\right)\left(\rho_{AB}\right)=\left(U⊗V\right)\rho_{AB}\left(U^{†}⊗V^{†}\right)$ for each state $\rho_{AB}$. Then,

(I⊗Φ)∘(U⊗V)=U⊗(Φ∘V)=(U⊗I)∘(I⊗(Φ∘V)).

Note that Φ∘V is still a Gaussian channel which sends $\rho_{B}$ to $\Phi (V\rho_{B}V^{†})$. Keep this in mind and let $\rho_{AB}$ be any (1+1)-mode Gaussian state. Then, there exists a local Gaussian unitary operation U⊗V such that $\sigma_{AB}=\left(U^{†}⊗V^{†}\right)\rho_{AB}\left(U⊗V\right)$ has CM of the standard form. By what we have proved above and Theorem 2, we see that

$$\begin{array}{cc} M(\left(I⊗\Phi \right)\rho_{AB})= & N_{F}^{G}\left(\left(I⊗\Phi \right)\left(\left(U⊗V\right)\sigma_{AB}\left(U^{†}⊗V^{†}\right)\right)\right)\\ = & M\left(\left(I⊗\Phi \right)\circ \left(U⊗V\right)\sigma_{AB}\right)=M\left(\left(U⊗I\right)\circ \left(I⊗\left(\Phi \circ V\right)\right)\sigma_{AB}\right)\\ = & M\left(\left(I⊗\left(\Phi \circ V\right)\right)\sigma_{AB}\right)\leq M\left(\sigma_{AB}\right)=M\left(\rho_{AB}\right), \end{array}$$

as desired, until now, we conclude that Equation (A1) holds for all (1+1)-mode Gaussian states. Following the same routine, let $\Phi_{B}=I$, one can show that

(A2) $$0\leq M\left(\left(\Phi_{A}⊗I\right)\rho_{AB}\right)\leq M\left(\rho_{AB}\right).$$

Combine Equations (A1) and (A2) together, it is clear that

$$\begin{array}{cc} M(\left(\Phi_{A}⊗\Phi_{B}\right)\rho_{AB})= & M(\left(I⊗\Phi_{B}\right)\circ \left(\Phi_{A}⊗I\right)\rho_{AB})\\ \leq & M(\left(\Phi_{A}⊗I\right)\rho_{AB})\\ \leq & M(\rho_{AB}), \end{array}$$

completing the proof. □

Our proof of Theorem 7 gives also another proof of Corollary 2. To do this, we need one more lemma on matrices.

**Lemma** **A2.**
*Let $B,K,M\in M_{n}\left(C\right)$ with B and M positive semidefinite. If both B and $KBK^{†}+M$ are invertible, then*

$$\begin{array}{c} K^{†}{\left(KBK^{†}+M\right)}^{-1}K\leq B^{-1}. \end{array}$$

*The equality holds if and only if M=0 and K is invertible.*

**Proof** **of** **Lemma** **A2.** Note that, if *A* and *B* are invertible, then $0\leq B\leq A\Leftrightarrow 0\leq A^{-1}\leq B^{-1}$. Assume that *K* is invertible. As $B\leq B+K^{-1}M{\left(K^{-1}\right)}^{†}$ we have

$$\begin{array}{c} K^{†}{\left(KBK^{†}+M\right)}^{-1}K={\left[K^{-1}\left(KBK^{†}+M\right){\left(K^{†}\right)}^{-1}\right]}^{-1}={\left(B+K^{-1}M{\left(K^{-1}\right)}^{†}\right)}^{-1}\leq B^{-1}, \end{array}$$

which reveals that the lemma is true for the case that *K* is invertible. Next, assume that *K* is not invertible. It is obvious that for sufficient small $\varepsilon_{0}>0$, K+εI is invertible for each $\varepsilon \in (0,\varepsilon_{0})$. As the set of all invertible matrices is an open subset in $M_{n}\left(C\right)$, the facts that $KBK^{†}+M$ is invertible and $\left(K+\varepsilon I\right)B{\left(K+\varepsilon I\right)}^{†}+M\to KBK^{†}+M$ as ε→0 entail that there is some $\varepsilon_{1}\in \left(0,\varepsilon_{0}\right)$ such that $\left(K+\varepsilon I\right)B{\left(K+\varepsilon I\right)}^{†}+M$ is invertible for all $\varepsilon \in (0,\varepsilon_{1})$. Thus, by what was proved above,

$${\left(K+\varepsilon I\right)}^{†}{\left(\left(K+\varepsilon I\right)B{\left(K+\varepsilon I\right)}^{†}+M\right)}^{-1}\left(K+\varepsilon I\right)\leq B^{-1}$$

holds for all $\varepsilon \in (0,\varepsilon_{1})$. Now, as $\lim_{\varepsilon \to 0}{\left(\left(K+\varepsilon I\right)B{\left(K+\varepsilon I\right)}^{†}+M\right)}^{-1}={\left(KBK^{†}+M\right)}^{-1}$, we see that

$$K^{†}{\left(KBK^{†}+M\right)}^{-1}K=\lim_{\varepsilon \to 0}{\left(K+\varepsilon I\right)}^{†}{\left(\left(K+\varepsilon I\right)B{\left(K+\varepsilon I\right)}^{†}+M\right)}^{-1}\left(K+\varepsilon I\right)\leq B^{-1}.$$

If the equality holds, that is, if $K^{†}{\left(KBK^{†}+M\right)}^{-1}K=B^{-1}$, then *K* is invertible and $B+K^{-1}M{\left(K^{-1}\right)}^{†}=B$, which entails that M=0. The converse is obvious, completing the proof. □

**Proof** **of** **Theorem** **7.** By the symmetry of M about the subsystems, we need only to prove that

$$M\left(\left(I⊗\Phi_{B}\right)\rho_{AB}\right)\leq M\left(\rho_{AB}\right)$$

holds for any Gaussian channel $\Phi_{B}$ performed on the subsystem *B* and any Gaussian state $\rho_{AB}$. Assume that the CM of $\rho_{AB}$ is $\Gamma =\left(\begin{array}{cc} A & C\\ C^{T} & B \end{array}\right)$ and $\Phi =\Phi_{B}=\Phi_{B}\left(K,M,\bar{d}\right)$. Then, by Equation (10), the CM of $\left(I⊗\Phi_{B}\right)\rho_{AB}$ is

$$\Gamma^{\prime }=\left(\begin{array}{cc} I & 0\\ 0 & K \end{array}\right)\left(\begin{array}{cc} A & C\\ C^{T} & B \end{array}\right)\left(\begin{array}{cc} I & 0\\ 0 & K^{T} \end{array}\right)+\left(\begin{array}{cc} 0 & 0\\ 0 & M \end{array}\right)=\left(\begin{array}{cc} A & CK^{T}\\ KC^{T} & KBK^{T}+M \end{array}\right).$$

Then

$$M\left(\left(I⊗\Phi \right)\rho_{AB}\right)=1-\frac{\det(\Gamma^{\prime })}{\det\left(A\right)\det\left(KBK^{T}+M\right)}=1-\frac{\det(A-CK^{T}{\left(KBK^{T}+M\right)}^{-1}KC^{T})}{\det(A)}.$$

Note that, as M≥0, by Lemma 2, we have

$$CK^{T}{\left(KBK^{T}+M\right)}^{-1}KC^{T}\leq CB^{-1}C^{T}.$$

This implies that

$$A-CK^{T}{\left(KBK^{T}+M\right)}^{-1}KC^{T}\geq A-CB^{-1}C^{T}$$

and thus $\det\left(A-CK^{T}{\left(KBK^{T}+M\right)}^{-1}KC^{T}\right)\geq \det\left(A-CB^{-1}C^{T}\right)$. It follows that

$$\begin{array}{cc} M(\left(I⊗\Phi \right)\rho_{AB})= & 1-\frac{\det(A-CK^{T}{\left(KBK^{T}+M\right)}^{-1}KC^{T})}{\det(A)}\\ \leq & 1-\frac{\det(A-CB^{-1}C^{T})}{\det(A)}=M\left(\rho_{AB}\right) \end{array}$$

as desired. □

**Proof** **of** **Equation** **(11).** According to the work in [38], the time revolution Markov main equation of a (1+1)-mode boson system in thermal environment is described by operator ${\bar{\Phi }}_{t}$:

$$\frac{d{\bar{\Phi }}_{t}}{dt}=\frac{i}{\hbar }\left[H,{\bar{\Phi }}_{t}\right]+\frac{1}{2h}\sum_{j}\left(V_{j}^{†}\left[{\bar{\Phi }}_{t},V_{j}\right]+\left[V_{j}^{†},{\bar{\Phi }}_{t}\right]V_{j}\right),$$

$$H=\sum_{k=1}^{2}\left(\frac{1}{2m_{k}}{\hat{P}}_{k}^{2}+\frac{m_{k}w_{k}^{2}}{2}{\hat{Q}}_{k}^{2}\right),$$

where $V_{j}=\sum_{k=1}^{2}a_{jk}{\hat{P}}_{k}+\sum_{k=1}^{2}b_{jk}{\hat{Q}}_{k},$ the combination coefficients $a_{jk}$, $b_{jk}$ are complex numbers with j=1,2,3,4 and k=1,2. Accordingly, $V_{j}^{†}=\sum_{k=1}^{2}a_{jk}^{*}{\hat{P}}_{k}+\sum_{k=1}^{2}b_{jk}^{*}{\hat{Q}}_{k},$ where $a_{jk}^{*}$ and $b_{jk}^{*}$ are conjugation of $a_{jk}$ and $b_{jk}$, respectively. For any (1+1)-mode Gaussian state $\rho_{AB}$ with covariance matrix Γ and mean *m*, by [38], the time revolution of the covariance matrix and mean are

(A3) $$\frac{dm(t)}{dt}=Ym\left(t\right),$$

(A4) $$\frac{d\Gamma (t)}{dt}=Y\Gamma \left(t\right)+\Gamma \left(t\right)Y^{T}+2D,$$

where

$$Y=\left(\begin{array}{cccc} -\lambda & \frac{1}{m_{1}} & 0 & 0\\ -m_{1}\omega_{1}^{2} & -\lambda & 0 & 0\\ 0 & 0 & -\lambda & \frac{1}{m_{2}}\\ 0 & 0 & -m_{2}\omega_{2}^{2} & -\lambda \end{array}\right)$$

is a 4×4 matrix, with λ the dissipation constant,

$$D=\left(\begin{array}{cccc} D_{{\hat{Q}}_{1}{\hat{Q}}_{1}} & D_{{\hat{Q}}_{1}{\hat{P}}_{1}} & D_{{\hat{Q}}_{1}{\hat{Q}}_{2}} & D_{{\hat{Q}}_{1}{\hat{P}}_{2}}\\ D_{{\hat{Q}}_{1}{\hat{P}}_{1}} & D_{{\hat{P}}_{1}{\hat{P}}_{1}} & D_{{\hat{Q}}_{2}{\hat{P}}_{1}} & D_{{\hat{P}}_{1}{\hat{P}}_{2}}\\ D_{{\hat{Q}}_{1}{\hat{Q}}_{2}} & D_{{\hat{Q}}_{2}{\hat{P}}_{1}} & D_{{\hat{Q}}_{2}{\hat{Q}}_{2}} & D_{{\hat{Q}}_{2}{\hat{P}}_{2}}\\ D_{{\hat{Q}}_{1}{\hat{P}}_{2}} & D_{{\hat{P}}_{1}{\hat{P}}_{2}} & D_{{\hat{Q}}_{2}{\hat{P}}_{2}} & D_{{\hat{P}}_{2}{\hat{P}}_{2}} \end{array}\right)$$

is diffusion matrix, and $D_{{\hat{Q}}_{k}{\hat{Q}}_{k}}=\frac{h}{2}\sum_{j=1}^{4}{\left|a_{jk}\right|}^{2}$, $D_{{\hat{P}}_{k}{\hat{P}}_{k}}=\frac{h}{2}\sum_{j=1}^{4}{\left|b_{jk}\right|}^{2}$, DQ^nP^m=h2Re∑j=14ajm*bjn, with k=1,2, m=1,2, and n=1,2. In addition, DQ^1Q^2=h2Re∑j=14aj1*aj2, DP^1P^2=h2Re∑j=14bj1*bj2. The solutions of differential Equations (A3) and (A4) can be found in [39]:

m(t)=exp(Yt)m(0),

(A5) Γ(t)=exp(Yt)[Γ(0)−Γ(∞)]exp(Yt)+Γ(∞),

where m(0) and Γ(0) stand for the mean and the covariance matrix of the initial state, and $\Gamma \left(\infty \right)=\lim_{t\to \infty }\Gamma \left(t\right)$. Furthermore, the covariance matrix satisfies

(A6) $$Y\Gamma \left(\infty \right)+\Gamma \left(\infty \right)Y^{T}=-2D.$$

For the convenience of computation, in the following, we assume the asymptotic state is Gibbs state. Then, the corresponding diffusion matrix is

$$D=\left(\begin{array}{cccc} \frac{\lambda }{2m_{1}\omega_{1}}\coth\frac{\omega_{1}}{2KT} & 0 & 0 & 0\\ 0 & \frac{\lambda m_{1}\omega_{1}}{2}\coth\frac{\omega_{1}}{2KT} & 0 & 0\\ 0 & 0 & \frac{\lambda }{2m_{2}\omega_{2}}\coth\frac{\omega_{2}}{2KT} & 0\\ 0 & 0 & 0 & \frac{\lambda m_{2}\omega_{2}}{2}\coth\frac{\omega_{2}}{2KT} \end{array}\right),$$

where *T* represents the temperature of the environment. Now, we prepare (1+1)-mode squeezed thermal state $\rho_{G}\left(0\right)$ as the initial state, with covariance matrix

$$\Gamma \left(0\right)=\left(\begin{array}{cccc} a & 0 & c & 0\\ 0 & a & 0 & -c\\ c & 0 & b & 0\\ 0 & -c & 0 & b \end{array}\right),$$

where a,b related to the compression parameters and the average photon number per mode, while *c* depends on the compression parameters and the average photon number on two modes. In order to capture the revolution behavior of the Gaussian state with time *t* and environment temperature *T*, we consider $\rho_{G}\left(t,T\right)$ as a function of *t* and *T*, and investigate the change trend of $\rho_{G}\left(t,T\right)$. Let a=b=2, c=1, $m_{1}=m_{2}=\omega_{1}=\omega_{2}=h=1$, and keep in mind that *K* is Boltzmann constant. Then,

(A7) $$Y=\left(\begin{array}{cccc} -1 & 1 & 0 & 0\\ -1 & -1 & 0 & 0\\ 0 & 0 & -1 & 1\\ 0 & 0 & -1 & -1 \end{array}\right)=\left(\begin{array}{cc} M & 0\\ 0 & M \end{array}\right),$$

(A8) $$D=\frac{1}{2}\coth\frac{1}{2KT}\left(\begin{array}{cccc} 1 & 0 & 0 & 0\\ 0 & 1 & 0 & 0\\ 0 & 0 & 1 & 0\\ 0 & 0 & 0 & 1 \end{array}\right)=\frac{1}{2}\coth\frac{1}{2KT}\left(\begin{array}{cc} I & 0\\ 0 & I \end{array}\right).$$

Substitute Equations (A7) and (A8) into Equation (A6), and denote $\Gamma \left(\infty \right)=\left(\begin{array}{cc} A & C\\ C^{T} & B \end{array}\right)$, one has

(A9) $$MA+AM^{T}=MB+BM^{T}=-\coth\frac{1}{2KT}I,$$

(A10) $$MC+CM^{T}=MC^{T}+C^{T}M^{T}=0.$$

Resolving Equations (A9) and (A10), we have

(A11) $$\Gamma \left(\infty \right)=D=\left(\begin{array}{cccc} \frac{1}{2}\coth\frac{1}{2KT} & 0 & 0 & 0\\ 0 & \frac{1}{2}\coth\frac{1}{2KT} & 0 & 0\\ 0 & 0 & \frac{1}{2}\coth\frac{1}{2KT} & 0\\ 0 & 0 & 0 & \frac{1}{2}\coth\frac{1}{2KT} \end{array}\right)=\frac{1}{2}\coth\frac{1}{2KT}I_{4}.$$

Substitute Equation (A11) into Equation (A5), one immediately gets the time revolution of the covariance matrix Γ(t,T) of $\rho_{G}\left(t,T\right)$ is of the form

$$\Gamma \left(t,T\right)=\left(\begin{array}{cccc} m(t,T) & 0 & 1 & 0\\ 0 & m(t,T) & 0 & -1\\ 1 & 0 & m(t,T) & 0\\ 0 & -1 & 0 & m(t,T) \end{array}\right),$$

where $m\left(t,T\right)=e^{-2t}\left(2-\frac{1}{2}\coth\frac{1}{2KT}\right)+\frac{1}{2}\coth\frac{1}{2KT}$. Therefore, we see that Equation (11) is true, completing the proof. □
